# Micro-Arc Oxidation Enhances the Blood Compatibility of Ultrafine-Grained Pure Titanium

**DOI:** 10.3390/ma10121446

**Published:** 2017-12-19

**Authors:** Lin Xu, Kun Zhang, Cong Wu, Xiaochun Lei, Jianning Ding, Xingling Shi, Chuncheng Liu

**Affiliations:** 1 Center of Micro/Nano Science and Technology, Jiangsu University, Zhenjiang 212013, China; 2211703075@stmail.ujs.edu.cn (K.Z.); 2211503099@stmail.ujs.edu.cn (C.W.); dingjn@ujs.edu.cn (J.D.); 2221703126@stmail.ujs.edu.cn (C.L.); 2 Laboratory Animal Research Center, Jiangsu University, Zhenjiang 212013, China; yuxin218@163.com; 3 Center of Photovoltaic Science and Engineering, Changzhou University, Changzhou 213164, China; 4 School of Materials Science and Engineering, Jiangsu University of Science and Technology, Zhenjiang 212013, China; shixingling1985@hotmail.com

**Keywords:** ultrafine-grained pure titanium, micro-arc oxidation, wettability, roughness, blood compatibility

## Abstract

Ultrafine-grained pure titanium prepared by equal-channel angular pressing has favorable mechanical performance and does not contain alloy elements that are toxic to the human body. It has potential clinical value in applications such as cardiac valve prostheses, vascular stents, and hip prostheses. To overcome the material’s inherent thrombogenicity, surface-coating modification is a crucial pathway to enhancing blood compatibility. An electrolyte solution of sodium silicate + sodium polyphosphate + calcium acetate and the micro-arc oxidation (MAO) technique were employed for in situ oxidation of an ultrafine-grained pure titanium surface. A porous coating with anatase- and rutile-phase TiO_2_ was generated and wettability and blood compatibility were examined. The results showed that, in comparison with ultrafine-grained pure titanium substrate, the MAO coating had a rougher surface, smaller contact angles for distilled water and higher surface energy. MAO modification effectively reduced the hemolysis rate; extended the dynamic coagulation time, prothrombin time (PT), and activated partial thromboplastin time (APTT); reduced the amount of platelet adhesion and the degree of deformation; and enhanced blood compatibility. In particular, the sample with an oxidation time of 9 min possessed the highest surface energy, largest PT and APTT values, smallest hemolysis rate, less platelet adhesion, a lesser degree of deformation, and more favorable blood compatibility. The MAO method can significantly enhance the blood compatibility of ultrafine-grained pure titanium, increasing its potential for practical applications.

## 1. Introduction

Biological materials used in applications involving direct contact with blood require favorable mechanical properties and exceptional blood compatibility. Titanium and titanium alloy (Ti-6Al-4V) are used in the repair and replacement of hard tissue, possess favorable mechanical performance and biocompatibility, and have crucial clinical value in applications such as prosthetic joints, artificial bone, and vascular stents [1,2]. Since Ti-6Al-4V possesses greater mechanical strength than titanium, it is the preferred material for hard tissue implants [3]. However, titanium alloy contains elements that are harmful to the human body, which may cause allergies, inflammation, and malignant reactions, limiting its further application in the field of biomedicine [4,5]. In recent years, distinct progress has been made in studies on ultrafine-grained titanium processed by equal-channel angular pressing (ECAP), which possesses favorable mechanical performance and does not contain elements that are toxic to the human body [6,7]. Ultrafine-grained titanium is a high-potential biological material [8] that can be applied in cardiac valve prostheses, vascular stents, and hip prostheses. In long-term implantation, however, ultrafine-grained titanium can produce thrombus because it is a metallic foreign body. When it comes into contact with blood, some thrombus is formed on the surface of the material, and plasma proteins and blood cells attach to its surface [9]. The surface of the metallic implant can be coated to perform composition and structural modification of the surface, thereby overcoming the thrombogenicity of the material and enhancing its blood compatibility. For instance, titanium nitride (TiN) film [10], diamond-like carbon film [11], and titanium oxide (TiO_2_) film [12] are often used as anticoagulant coatings. Among these, TiO_2_ coating can effectively inhibit the formation of thrombus on the surface of titanium material and enhance its blood compatibility [13]. However, the TiO_2_ protective coating formed from the natural passivation of titanium material is exceedingly thin. After an external material is implanted in the human body for long-term service, under gradual external wear and corrosion in body fluids, thrombus may form once the passivated TiO_2_ coating is damaged. This results in the loosening or fracturing of the implant and eventually the failure of the replacement. Therefore, the blood compatibility of titanium material can be improved through a suitable increase in the thickness of the TiO_2_ protective coating and the activation of this coating on the titanium surface [14,15].

The surface modification of titanium material to obtain favorable comprehensive performance is therefore necessary and enhances the biological properties of the material to satisfy clinical needs. Sunny et al. [16] used anodic oxidation to prepare thin Ti-O film and found that as the film’s thickness increased, the ratio of fibrinogen to albumin that attached to the surface decreased and blood compatibility improved significantly. Huang et al. [17] found that TiO_2_ with rutile structure showed a distinct improvement in blood compatibility following an increase in thickness. Furthermore, Lu et al. [18] and Feng et al. [19] have successfully prepared a superhydrophilic surface that enables titanium to demonstrate more favorable blood compatibility. Generally, such a surface can be obtained by controlling roughness (micron- or nanoscale) to enhance surface energy. Surface roughness can be controlled through electrochemical deposition [20], the sol-gel method [21], and anodic oxidation [22]. Among these, the new surface modification technology of micro-arc oxidation (MAO) can be used to grow TiO_2_-based rough and porous ceramic coating [23,24] and can dope the coating with bioactive elements, such as calcium and phosphate, by adjusting electrolyte composition to further enhance the blood compatibility of the implant. As reported by Wang et al. [25] more Ca and P in the MAO-treated coating on Ti can improve the blood compatibility when compared with the MAO-treated coating containing small amounts of Ca and P. At present, further research is required on the use of MAO parameter optimization to build the surface microstructure of ultrafine-grained pure titanium and, thus, enhance the blood compatibility of the material.

This paper proposes an electrolyte solution of sodium silicate + sodium polyphosphate + calcium acetate. MAO technology was employed in the bioactive modification of an ultrafine-grained pure titanium material. Subsequently, a TiO_2_-based rough and porous ceramic coating was prepared to enable observation of the effects of oxidation time and voltage on the surface topography, roughness, and wettability of the coating. The blood compatibility of the material was studied through tests of the hemolysis rate, the time required for dynamic coagulation, and platelet adhesion. These experiments tested the feasibility and safety of the biomaterials for clinical use, to serve as a theoretical reference and technical support for further medical research.

## 2. Materials and Methods

### 2.1. Preparation of the Coating

In the test, commercial pure titanium (Grade 2) specimens with the element composition of 0.16 O, 0.02 N, 0.02 C, 0.001 H, 0.14 Fe, and the balance Ti (wt%) were used as raw materials for ECAP processing to obtain ultrafine-grained pure titanium [7]. The substrate was cut into round wafers measuring Φ 18 mm × 2 mm, and polished with SiC abrasive papers from 150 to 5000 grit. This was followed by ultrasonic cleaning using acetone, alcohol, and deionized water. A JHMAO-380/20A MAO device (Xi’an Jin Tang Material Application Technology Co., Ltd., Xi’an, China) was employed at constant current mode, adopting an electric parameter with an impulse frequency of 400 Hz, current density of 20 A/dm^2^, duty cycle of 10%, and oxidation times of 3 min, 6 min, 9 min, 12 min, and 15 min to perform MAO. Ultrafine-grained pure titanium, as the experimental substrate, was denoted as E00, and samples treated for 3 min, 6 min, 9 min, 12 min, and 15 min were designated as series E: E03, E06, E09, E12 and E15, respectively. The electrolyte solution were prepared from deionized water and a composition of 10 g/L sodium silicate, 8 g/L sodium polyphosphate, and 20 g/L calcium acetate (all analytically pure). After the MAO test sample was processed through ultrasound, the sample was dried and set aside.

### 2.2. Surface Characterization

The surface morphology of the MAO coating was observed using a field emission scanning electron microscope (SEM, JEOL JSM-7001F, Tokyo, Japan) with an energy dispersive X-ray spectrometer (EDS, Oxford INCA) for chemical analysis. High voltage was 15 kV and the work distance was about 10 mm. The thickness of the coatings was measured by TT260 coating thickness gauge. A D8-ADVANCE X-ray diffractometer (Bruker Company, Karlsruhe, Germany), Cu target Kα alpha radiation (incident wavelength 0.154056 nm) was used, and the scanning speed was 4°/min. A step width was 0.01° in a scanning range between 10° and 80° was used to analyze the phase composition of the coating. Micropore parameters of the coatings were analyzed by using MATLAB image processing technology. A ContourGT non-contact 3D profiler (Bruker Company, Madison, WI, USA) was used to measure the surface roughness. From each test sample, 10 different areas were randomly selected for measurement, and the resulting roughness (*R*a) values were averaged. A DataPhysics OCA25 video optical contact angle measurement instrument and the pendant drop method were employed to measure the static contact angle of distilled water and ethylene glycol with a droplet of 2 μL on the test sample surface, and the Owens [26] two-liquid method was used to calculate the surface energy.

### 2.3. Hemolysis Test

In the hemolysis test, 8 mL of fresh anticoagulated whole rabbit blood was extracted, with an additional 10 mL 0.9% sodium chloride solution (i.e., saline solution) at a volume ratio of 4:5 to obtain a dilution of anticoagulated whole rabbit blood. Six samples for the experiment (substrate and another five samples treated for 3 min, 6 min, 9 min, 12 min, and 15 min, respectively) were separately placed in different sterile centrifuge tubes with 10 mL 0.9% sodium chloride solution, and then in 37 °C water baths at a constant temperature for 30 min. Under the same experimental conditions, the positive control used 10 mL of distilled water and 0.2 mL of diluted anticoagulated whole rabbit blood, and the negative control used 10 mL 0.9% sodium chloride solution and 0.2 mL of diluted anticoagulated whole rabbit blood. These were slowly mixed, and the test tubes were placed once again into a 37 °C water bath for 60 min. Subsequently, these were centrifuged on a centrifugal machine at 850 r/min for 5 min. The supernatant fluid was drawn and transferred into a cuvette; the absorbance of the supernatant was measured using a spectrophotometer at a wavelength of 545 nm. Each sample was subjected to three parallel tests and the mean was obtained. The hemolysis rate was calculated according to the following equation:

(1) $$HR\left(\%\right)=\frac{D_{t}-D_{nc}}{D_{pc}-D_{nc}}\times 100\%$$

where $D_{t}$ is the sample absorbance, $D_{\mathrm{pc}}$ is the positive control group absorbance, and $D_{\mathrm{nc}}$ is the negative control group absorbance.

### 2.4. Test of Dynamic Coagulation Time

A total of 30 μL of the fresh anticoagulated rabbit blood was separately dropped onto the sample surfaces and allowed to stand for 10 min, 20 min, 30 min, 40 min, and 50 min. Clamps were then used to immediately place samples gently into a centrifugal tube containing 15 mL of distilled water and allowed to stand for 10 min. Once the uncoagulated erythrocytes had fully dissolved in the water, a spectrophotometer was used at a 540 nm wavelength to test the absorbance. Distilled water was used as a blank control. Each sample was subjected to three parallel tests and the mean was obtained to draw a relationship curve of absorbance and time.

### 2.5. Platelet Adhesion Test

First, 50 mL of anticoagulated whole rabbit blood was collected and centrifuged on a centrifugal machine at 1000 r/min for 10 min. The upper portion of the platelet-rich plasma was extracted and prepared, and the test sample awaiting testing was immersed in the platelet-rich plasma. After 120 min of incubation in a 37 °C constant temperature water bath, the test sample was removed. Saline solution was used to rinse unattached platelets on the surface, and the sample was set in 2.5% glutaraldehyde solution for 12 h. The sample was removed and rinsed again with saline solution, and ethanol solution (at concentrations of 30%, 50%, 70%, 80%, 90%, and 100%) was used separately for gradient dehydration for 10–15 min each time. Next, tert-butyl alcohol was used for dehydration twice, 10–15 min each time, by using CO_2_ critical-point drying followed by metal spraying. The sample was placed under a scanning electron microscope (SEM) to observe the changes in platelet adhesion. From each sample, five different observation sites were selected, and the images were saved.

### 2.6. Prothrombin Time (PT) and Activated Partial Thromboplastin Time (APTT) Tests

First, 2 mL of fresh anticoagulated whole rabbit blood was extracted and centrifuged at a speed of 900 r/min for 10 min to obtain the upper layer of platelet-rich plasma. A sample preheated in a 37 °C incubator was placed in the platelet plasma and incubated was continued for 2 min. An automated blood coagulation analyzer was used to measure and record PT and APTT. To ensure the accuracy of the test data, the test was conducted three times with each group and the mean was calculated.

## 3. Results and Discussion

### 3.1. Microscopic Topography and Elemental Compositions of Oxidation Coating

**Figure 1:**
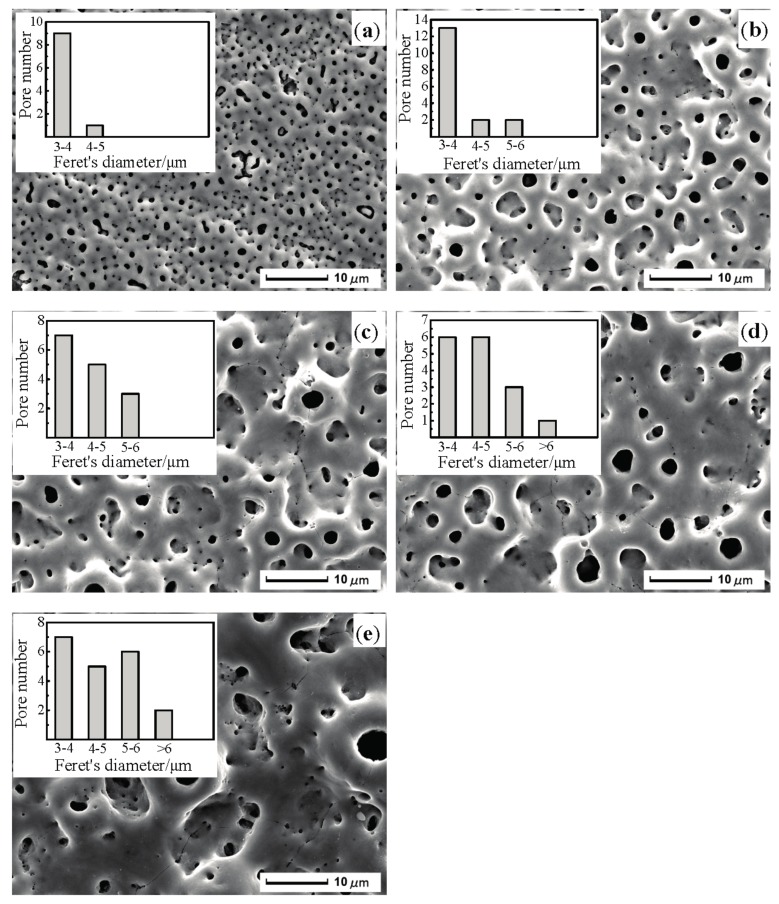
SEM (scanning electron microscope) morphology and statistical data of micropores with diameter exceeding 3 μm of the MAO (micro-arc oxidation) coating at different oxidation times: (**a**) 3 min; (**b**) 6 min; (**c**) 9 min; (**d**) 12 min; and (**e**) 15 min.

Figure 1 shows the microscopic topography and statistical data of micropores with diameters exceeding 3 μm of the MAO coating on ultrafine-grained pure titanium at different oxidation times. As shown in Figure 1a, at an oxidation time of 3 min, a large number of microscopic pores were distributed on the coating surface, and the pore sizes were still below 5 µm. The surface was relatively flat and compact, and the groove marks remaining from the sanding of the substrate were clearly visible. These microscopic pores were due to a reaction between the substrate and electrolyte solution in micro-discharge channels during MAO that formed oxide discharge channels. Figure 1b indicates that, following the extension of the oxidation time to 6 min, there were some micropores whose apertures were greater than 5 µm, and pore sizes on the coating surface increased, whereas the number of pores decreased. Several inconsistently-sized, raised volcanic hill shapes were distributed around the oxide discharge channels, and the coating surface gradually became rough. Following the increase in oxidation time, the pore size on the coating surface rapidly increased, the topographic characteristic of a “crater” formed by molten oxide was more pronounced, and the number of micropores further decreased (Figure 1c).

Figure 1d,e indicate that as oxidation time was extended, pore sizes on the coating surface continuously increased and the number of pores continuously decreased; the distribution became uneven and micropores were nested in large pores. As the MAO treatment time was extended to 12 min, there were some micropores whose aperture was greater than 6 µm. When the treatment time increased to 15 min, the number of pores with a diameter of 5–6 µm increased obviously, and the number of pores with a diameter of 6 μm also gradually increased.

**Figure 2:**
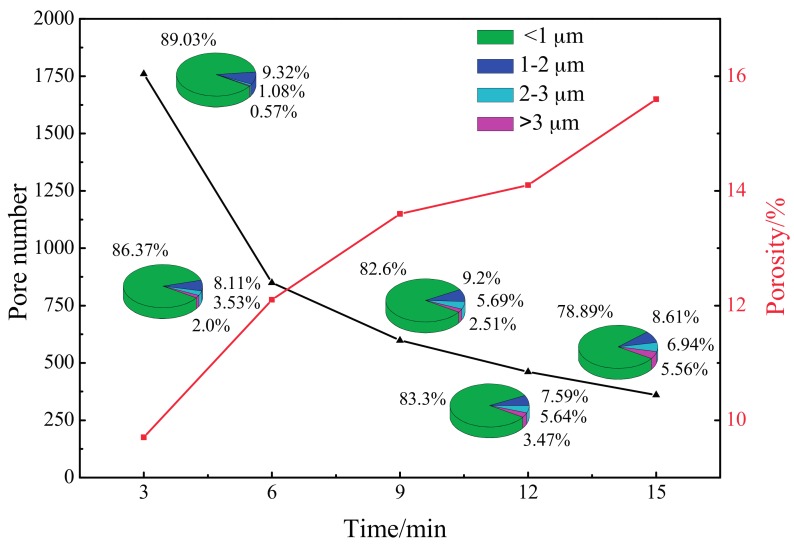
Statistical data of all micropores and porosity on MAO coating at different oxidation times.

As shown in Figure 2, with increasing oxidation time, the total number of micropores decreased greatly. After 3 min of MAO treatment, the percentage of micropores whose aperture was less than 1 µm was approximately 90%; as the oxidation time increased to 6 min, the proportion of micropores less than 1 µm in diameter decreased slightly. By contrast, the proportion of micropores whose aperture was 2–3 µm and greater than 3 µm went up. For the MAO coating treated for 15 min, the quantity of micropores whose aperture was less than 4 µm was at the minimum; nevertheless, the proportion of micropores of 2 µm and above in diameter increased continuously. In addition, the porosity increased significantly, with the values being 9.7%, 12.1%, 13.6%, 14.1%, and 15.6% for the five oxidation times.

Table 1 shows elemental compositions of the MAO coating surfaces. It was observed that the basic compositions of the coating surface were Ti, O, Si, Ca, and P elements, which indicated that Si, Ca, and P elements were successfully incorporated into the coating due to the action of micro-arc discharge.

### 3.2. Variation of Oxidation Coating Thickness and Voltage

**Figure 3:**
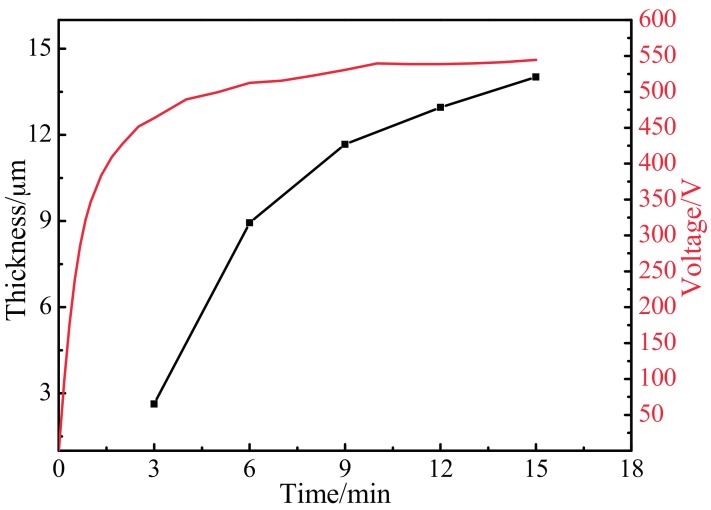
Variation of MAO coating thickness and voltage at different oxidation times.

Figure 3 shows the variation of the MAO coating thickness and voltage at different oxidation times. From the voltage-time curve, in the initial stage of oxidation (*t* < 2 min), the voltage rapidly increased to 450 V and, subsequently, the growth rate of the voltage reduced obviously with the oxidation process. After 10 min of oxidation, the voltage tended to a relatively stable value—about 520 V. It is obvious that the thickness gradually increased with increases in the oxidation time and voltage. To be more specific, the thickness of the coatings was 2.63 µm, 8.94 µm, 11.67 µm, 12.96 µm, and 14.02 µm respectively, at certain oxidation times. Although the coating thickness increased with the oxidation time, the rate of thickening decreased. A comprehensive analysis reveals that this was because the coating was thinner and the arcing voltage of MAO was low during early oxidation; discharge sparks were small, and puncturing the oxidation coating was relatively easy, which resulted in being prone to breakdown and the rapid growth of the oxidized coating. Moreover, the molten oxide overflowed through the discharge channel, cooled rapidly, and was deposited after reaching the surface. With increases in the oxidation time, voltage, and energy, repeated breakdown made the oxide coating thicker. When the oxidation time was extended to 6 min, following the increase in coating thickness, the discharge resistance increased. The thicker the coating was, the more difficult the electric breakdown became. When the oxidation time was greater than 9 min, because the coating thickness was further increased, puncturing the coating became increasingly difficult and the rate of thickening continued to decrease as a result. Thus, the growth rate of the coating thickness gradually reduced with the accretion of the coating.

### 3.3. XRD Pattern of the Oxidation Coating

**Figure 4:**
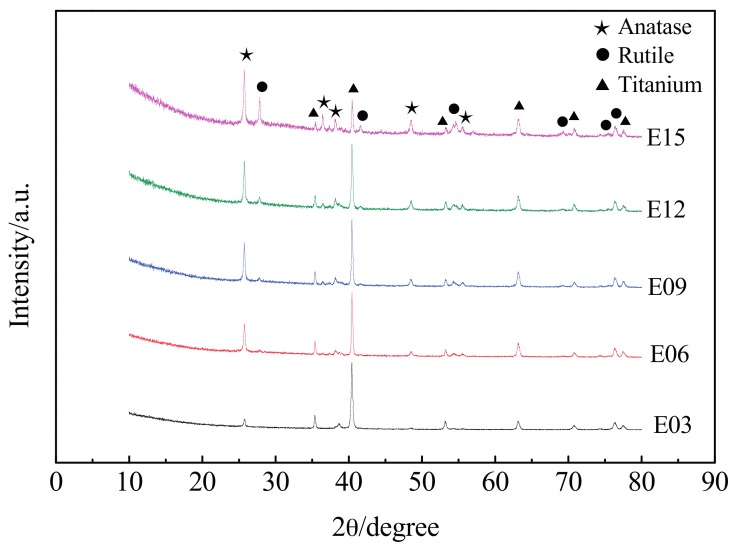
XRD of MAO coating at different oxidation times. E03: 3 min; E06: 6 min; E09: 9 min; E12: 12 min; and E15: 15 min.

Figure 4 shows the XRD patterns of MAO coating at different oxidation times. They were relatively similar and the diffraction peaks of the Ti, anatase-phase TiO_2_, and rutile-phase TiO_2_ appeared in all samples. Among these, the diffraction peaks of Ti were derived from the ultrafine-grained titanium substrate. Sicne the MAO coating had a porous structure, an X-ray diffractometer could detect the substrate. The Ti phase was evident. TiO_2_ has three main crystalline phases: rutile (tetragonal), anatase (tetragonal), and brookite (orthorhombic). However, rutile and anatase play a more important role than brookite in the application of TiO_2_, and the research interests of surface science and technology are mainly on rutile and anatase. The rutile phase was the most stable under higher temperatures, and the anatase phase formed at a relatively low temperature, transforming into the rutile phase at temperatures higher than 800 °C [27]. The quality scores of the rutile phase can be calculated according to the following equation [28]:

(2) $$W_{R}\;=\;1/(1\;+\;0.8I_{A}/I_{R})$$

where $I_{R}$ and $I_{A}$ are the X-ray diffraction peak strengths of the rutile-phase (110) and anatase-phase (101) crystal planes, respectively.

**Figure 5:**
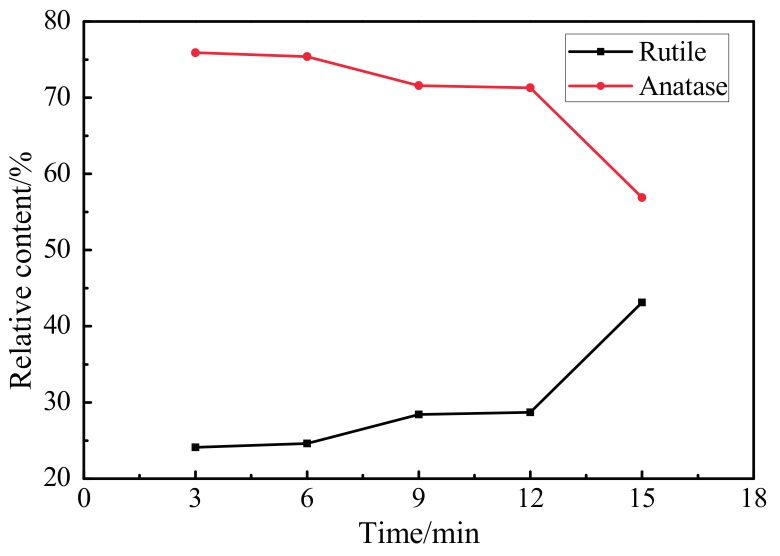
Relative contents (%) of rutile and anatase phases in MAO coating at different oxidation times.

Figure 5 shows relative contents (%) of rutile and anatase phases in MAO coating at different oxidation times. When the oxidation time was 3 min, the diffraction peak strength of the ultrafine-grained pure titanium substrate was larger because of the shorter reaction time. The peaks of the anatase- and rutile-phase TiO_2_ were relatively small; the content of the rutile-phase TiO_2_ was only 24.1%. Following the extension of oxidation time, the diffraction peak strengths of the anatase- and rutile-phase TiO_2_ in the oxidation layer gradually increased; the diffraction strength of the Ti diffraction peak, derived from the ultrafine-grained pure titanium substrate, gradually weakened; and the content of the rutile phase gradually increased. These results were because, with the increase in oxidation time, the temperature and pressure generated in the MAO reaction continuously increased, the metastable-state anatase-phase TiO_2_ continuously transformed into steady-state rutile-phase TiO_2_ under high temperatures, and the coating continuously thickened. Therefore, at an oxidation time of 15 min, the rutile-phase TiO_2_ in the MAO coating reached a maximum value of 43.1%.

### 3.4. Oxidation Coating Roughness

**Figure 6:**
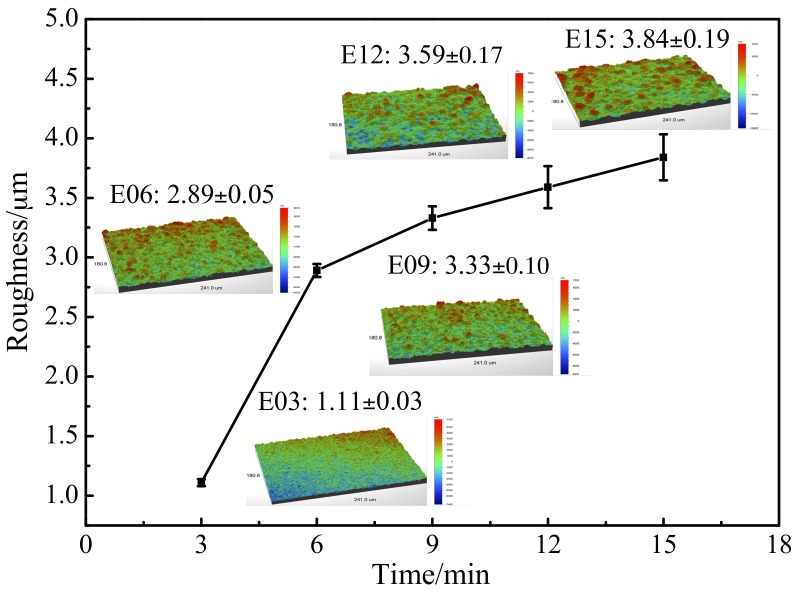
Roughness-time curve of MAO coating at different oxidation times. E03: 3 min; E06: 6 min; E09: 9 min; E12: 12 min; and E15: 15 min.

Figure 6 shows the relationship curve between oxidation time and MAO coating roughness. When other parameters were the same, the roughness of the coating increased following increases in oxidation time. This was because, with the increase in time, the surface pores of the oxidation coating became enlarged and the molten oxide around the pores increased, causing an increase in surface roughness. Roughness gradually increased from its initial 1.11 µm at 3 min, reaching 2.89 µm at 6 min, and finally 3.84 µm at 15 min. The roughness of the oxidation coating changed more rapidly during the early stages of the MAO reaction, and changes in roughness gradually slowed during later stages. This was because, during the initial stages of the MAO reaction, the coating was in a rapid growth stage and large amounts of molten mixture accumulated, causing the roughness of the oxidation coating at this time to increase rapidly. Once the MAO reaction had been executed for a certain length of time, the coating entered a slow growth stage, and in addition to accumulating around the pores, the molten mixture filled the pores; this made the roughness changes at this time less evident than before. Previous studies have shown that material with some ranges of surface roughness possess more favorable anticoagulant performance [29,30].

### 3.5. Coating Surface Wettability

To study the wettability of ultrafine-grained pure titanium and MAO coating on ultrafine-grained pure titanium at different oxidation times, their surface energy was calculated by measuring the contact angles of different liquids on each sample surface. The contact angle θ is the primary criterion for measuring the wetting degree of liquids on solid surfaces; a smaller contact angle indicates a larger surface energy and more favorable wettability.

**Figure 7:**
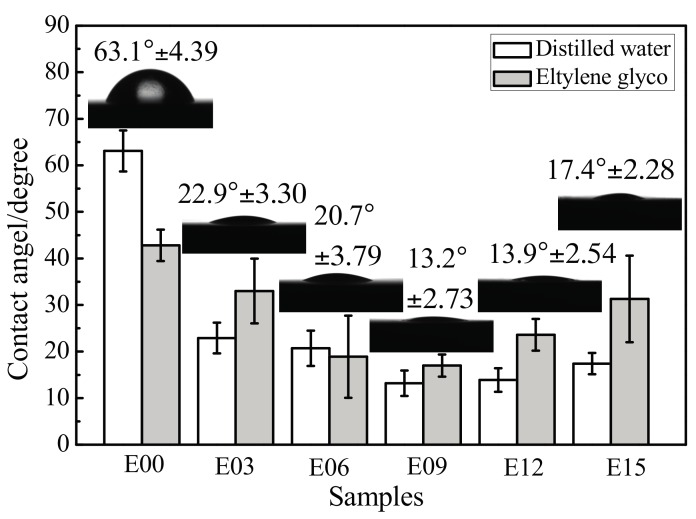
Contact angles of distilled water and ethylene glycol on sample surfaces and the forms of distilled water droplets on different sample surfaces. E00: substrate; E03: 3 min; E06: 6 min; E09: 9 min; E12: 12 min; and E15: 15 min.

Figure 7 shows the contact angle measurements of each sample surface with distilled water and ethylene glycol, as well as the forms of distilled water droplets on different sample surfaces; the black portions underneath the water droplets are the samples. Regardless of whether the tested liquid was distilled water or ethylene glycol, after MAO processing, the contact angles of the coating surface all decreased in comparison with the ultrafine-grained pure titanium substrate, and hydrophilic performance improved significantly. In particular, when the oxidation time was 9 min, the contact angles decreased from 63.1° and 43° to 13.2° and 17°, respectively, because the rough and porous surface structure possessed increased water retention capacity after MAO.

As demonstrated in Figure 7, the droplets on the substrate surfaces were nearly hemispherical. After MAO processing, the droplets were more able to spread over the ultrafine-grained pure-titanium MAO coating surface. This indicated that MAO processing reduced the contact angles of the sample surfaces and could more favorably enhanced their wettability.

According to the determined test liquids (distilled water and ethylene glycol) in ultrafine-grained pure titanium and the contact angles of the MAO coating surface at different oxidation times, as well as the surface energy parameters (as Table 1) of these test liquids, the Owens two-liquid method was used to calculate the surface energy of the samples:

(3) $$\gamma_{L}\left(1\;+\;\cos\theta \right)\;=\;2(\gamma_{S}^{D}\cdot \gamma_{L}^{D}{)}^{\frac{1}{2}}\;+\;2(\gamma_{S}^{P}\cdot \gamma_{L}^{P}{)}^{\frac{1}{2}}$$

(4) $$\gamma_{S}\;=\;\gamma_{S}^{D}\;+\;\gamma_{S}^{P}$$

where θ is the contact angle of the test liquid on the solid surface, $\gamma_{L}^{D}$ is the dispersion force component of the test liquids, $\gamma_{L}^{P}$ is the polar force component of the liquids, and $\gamma_{L}$ is the surface energy of the liquids. $\gamma_{S}^{D}$ and $\gamma_{S}^{P}$ are the dispersion and polar force of the solid surface, respectively, and $\gamma_{S}$ is the solid surface energy.

**Figure 8:**
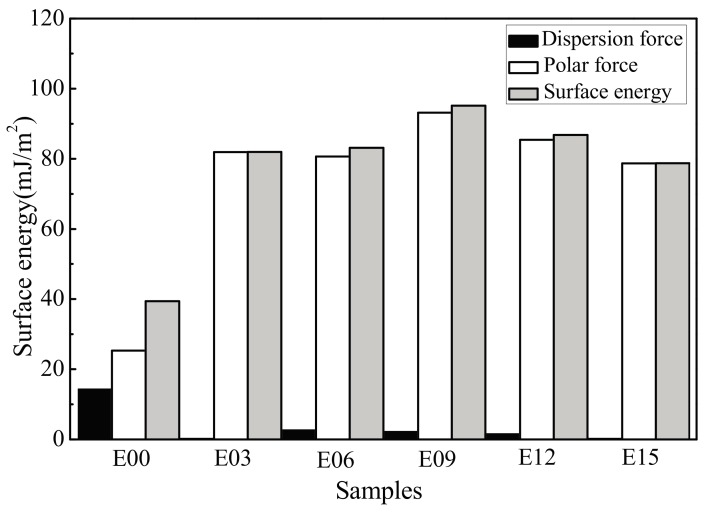
Surface energy of samples. E00: substrate; E03: 3 min; E06: 6 min; E09: 9 min; E12: 12 min; and E15: 15 min.

By respectively substituting the surface energy parameters and measured contact angle data of distilled water and ethylene glycol in Table 2 into Equations (3) and (4), a set of simultaneous equations for the surface energy of the samples can be obtained. The results are shown in Figure 8. After MAO processing, the surface energies of the sample surfaces all increased significantly. In particular, at an oxidation time of 9 min, the coating surface energy rose from 38.31 mJ/m^2^ to 92.65 mJ/m^2^, an increase of 141.8%. The change sharply differed in the dispersion and polar forces in surface energy. The dispersion force component of the sample surface after MAO processing was distinctly reduced in comparison to that of the substrate, and the polar force component had greatly increased. At an oxidation time of 9 min, the dispersion force component decreased from 13.72 mJ/m^2^ to 1.97 mJ/m^2^ and the polar force component increased from 24.59 mJ/m^2^ to 90.69 mJ/m^2^. This indicated that the MAO processing had a greater effect on the polar force component; the significant increase in surface energy was primarily due to the increase in its polar force component. Possible causes may have been: (1) after MAO processing, the large amount of micro/nanopores formed on the oxidation coating surface caused its specific surface area to increase, which benefited water retention; (2) MAO processing resulted in an uneven coating surface, increased roughness, increased adsorbability, and decreased contact angles, all of which together affected the surface energy; and (3) the −OH^−^ and −O^2−^ oxygen-containing groups formed on the coating surface effectively introduced hydrophilic groups, changing the number of polar groups on the coating surface and causing surface polarity to increase, resulting in an increase in the surface energy.

### 3.6. Hemolysis Rate

**Figure 9:**
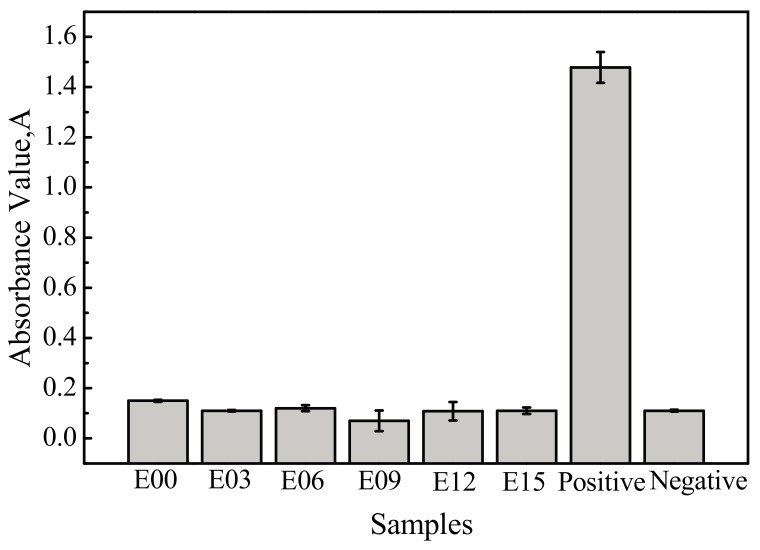
Absorbance value of erythrocyte on the sample surfaces. E00: substrate; E03: 3 min; E06: 6 min; E09: 9 min; E12: 12 min; and E15: 15 min.

Figure 9 shows the measured absorbance values for the ultrafine-grained pure titanium surfaces and the MAO coating at different oxidation times (3 min, 6 min, 9 min, 12 min, and 15 min). The absorbance values of the positive and negative control groups were 1.478 and 0.11, respectively, meeting national standards. The absorbance values of the sample surfaces before and after MAO did not change greatly, and the absorbance value of the coating was the smallest at an oxidation time of 9 min, at 0.07.

**Figure 10:**
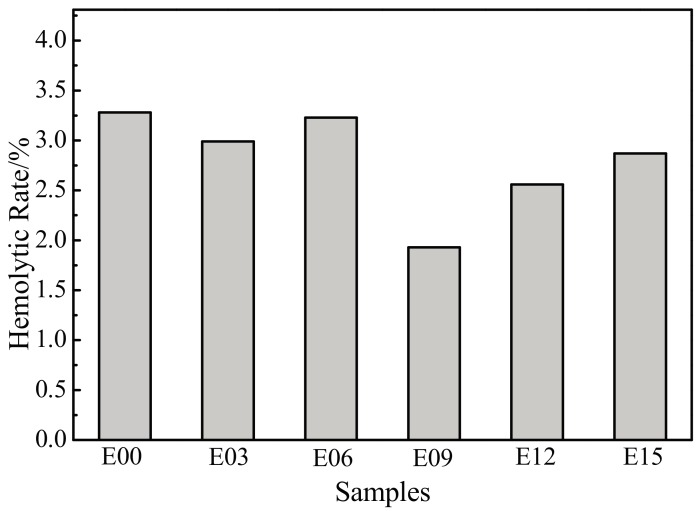
Hemolysis rate of samples. E00: substrate; E03: 3 min; E06: 6 min; E09: 9 min; E12: 12 min; and E15: 15 min.

The measured absorbance values were substituted into Equation (1) to calculate hemolysis rates, as shown in Figure 10. The hemolysis rate of each sample was much lower than 5%, and all were in accordance with the hemolysis rate requirements for medical materials that do not come in direct blood contact and would not generate hemolysis. In comparison with the 3.28% hemolysis rate of the unmodified titanium substrate, the hemolysis rate of each sample decreased after MAO processing. This indicated that the modification reduced the destruction of erythrocytes by samples and that the antihemolysis performance of the samples improved. Moreover, when the oxidation time was 9 min, the hemolysis rate was the lowest at only 1.93%; the antihemolysis performance at this time was the most favorable.

### 3.7. Dynamic Coagulation Time

**Figure 11:**
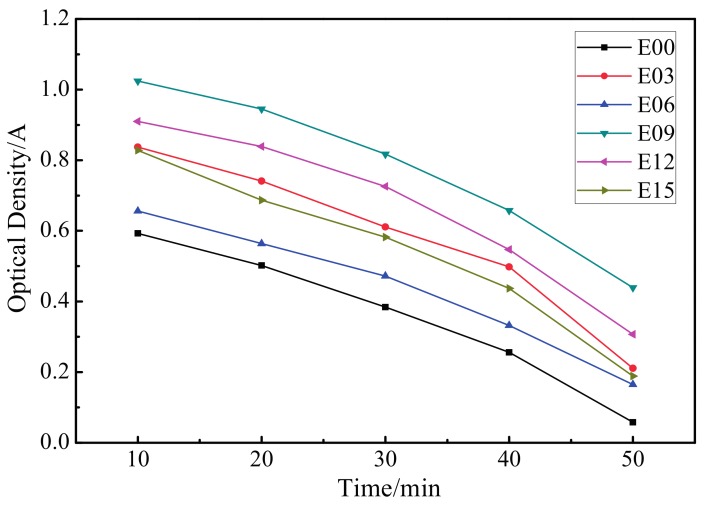
Blood coagulation profiles of samples. E00: substrate; E03: 3 min; E06: 6 min; E09: 9 min; E12: 12 min; and E15: 15 min.

Figure 11 presents the dynamic coagulation time curve for the ultrafine-grained pure titanium surfaces and the MAO coating at different oxidation times (3 min, 6 min, 9 min, 12 min, and 15 min). The absorbance value can be used to represent the relative amount of uncoagulated blood on the surface after contact between the material and blood. A smaller absorbance value implied increases in the relative amount of coagulated blood and the degree of coagulation.

Additionally, the absorbance of titanium substrates was at its minimum in each period, which indicated that the amount of coagulated blood was simultaneously at its maximum. After MAO processing, the absorbance value of each sample was larger than that of the substrate, indicating that modification enhanced the absorbance of the substrate, increased the relative amount of uncoagulated blood, extended the dynamic coagulation time, and improved blood compatibility. Among these, the absorbance value of the sample that was oxidized for 9 min was highest; its coagulation time was the longest, the degree of activation of the coagulation factor was the lowest, and antihemolysis performance was the most favorable.

### 3.8. Prothrombin Time and Activated Partial Thromboplastin Time

Table 3 shows the PT and APTT times of each sample before and after MAO. Increases in PT and APTT values resulted in decreased coagulation factors in the coagulation system, implying improved antihemolysis [31]. At different oxidation times, the PT and APTT values of the MAO coating were all within normal ranges. These values obtained from testing demonstrated an increase followed by a decrease as the oxidation time increased, which indicated that the MAO time affected the antihemolysis of ultrafine-grained pure titanium. After MAO, the PT and APTT values of ultrafine-grained pure titanium were all higher than those of the substrate, indicating that MAO processing could enhance its anticoagulant property. At an oxidation time of 9 min, the PT and APTT values of the MAO coating were at their maximum; the coagulation factor was the lowest; and the anticoagulant property was the most favorable.

### 3.9. Platelet Adhesion Behavior

**Figure 12:**
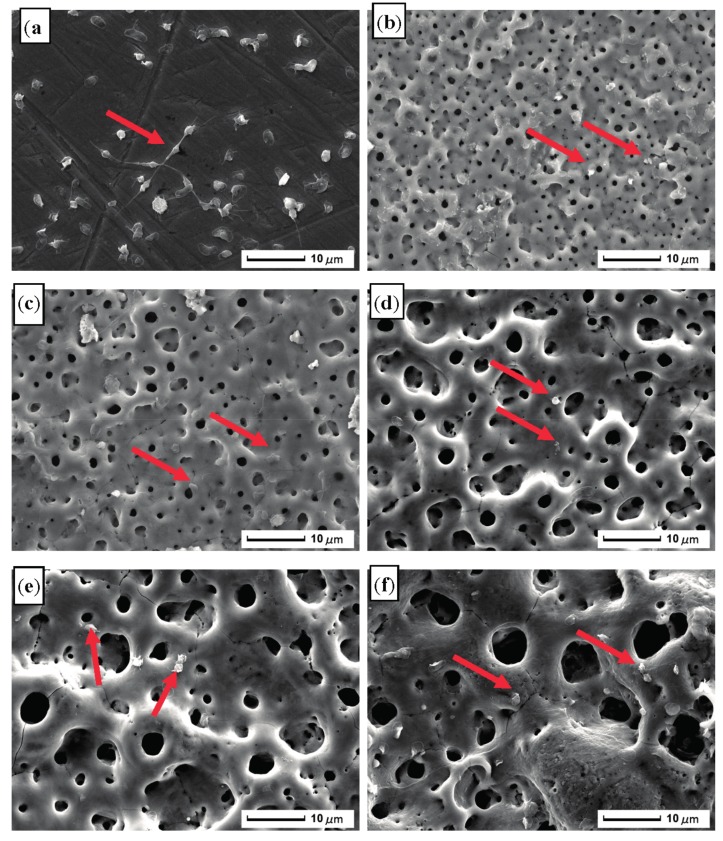
SEM images of the adhesion deformation of platelets on the sample surfaces: (**a**) E00: substrate; (**b**) E03: 3 min; (**c**) E06: 6 min; (**d**) E09: 9 min; (**e**) E12: 12 min; and (**f**) E15: 15 min.

Figure 12 presents the SEM images of the deformation of platelet adhesion for the ultrafine-grained pure titanium surfaces and the MAO coating at different oxidation times (3 min, 6 min, 9 min, 12 min, and 15 min). Each sample showed platelet adhesion (indicated by the red arrow in the figure). The highest number of platelets adhered to the surface of the titanium substrate, and platelet aggregation formed and extended pseudopodia, connecting into a mesh; the deformation was more evident. After MAO processing, the number of adhered platelets on the sample surfaces clearly decreased, the degree of platelet deformation was reduced, and platelets on some samples extended a few dendritic pseudopodia deeply into the pores. In particular, in the sample oxidized for 9 min, the lowest number of platelets adhered, aggregation did not occur, and the degree of deformation was minimal; essentially, the platelets were not activated.

**Figure 13:**
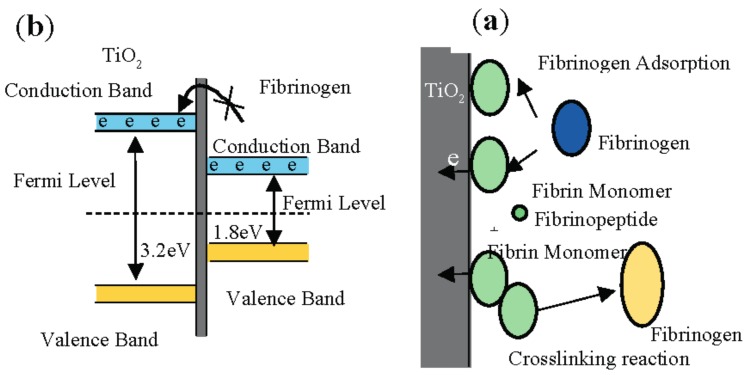
Schematic diagram for electrochemical action of TiO_2_ and fibrinogen after adsorption: (**a**) electrochemical process; and (**b**) energy band diagram [33,35].

In comparison to the substrate, the MAO coating surface possessed more favorable performance in preventing platelet adhesion and deformation. This was primarily because the adhesion of platelets on a material surface is closely related to the types, numbers, and 3D conformation changes of the plasma proteins adsorbed by the material surface, primarily albumin and fibrinogen. When the protein layer adsorbed by the surface of the material was albumin, the material surface demonstrated biopassivation, reducing the adhesion of platelets on the surface and preventing coagulation [32], as shown in Figure 13a. Sawyer et al. [33] proposed an electrochemical hypothesis, finding that the electronic structure of fibrinogen was similar to that of intrinsic semiconductors. The forbidden band gap was narrow, measuring 1.8 eV. After fibrinogen was adsorbed on the material surface, its valence electrons were transferred to the material surface it was in contact with. This caused the conformational change of the fibrinogen and decompose the fibrinogen into fibrin monomers and fibrinopeptides, followed by polymerization and cross-linking between monomers to form an intermediate polymer, accelerating the coagulation process. Thus, to prevent the transfer of the electric charge direction and the material it was in contact with, the material must possess a smaller work function [34]. After the ultrafine-grained pure titanium substrate underwent MAO modification, it formed a porous ceramic coating with the mixed crystal structures of anatase- and rutile-phase TiO_2_ as its primary body. As shown in Figure 13b, TiO_2_ possessed the characteristics of wide forbidden-band-gap semiconductors, with a gap of 3.2 eV. The conduction and forbidden bands of fibrinogen were located in the forbidden band gap of TiO_2_, and electrons were in the conduction band of TiO_2_. This inhibited the transfer of electrons from the TiO_2_ coating surface by the adsorbed fibrinogen and was beneficial in maintaining the normal fibrinogen conformation; it was, thus, more difficult for platelets to aggregate on the TiO_2_ coating surface and deform.

Researchers have attempted to explain the adsorption of fibrin by using the pathways of thermodynamics. Since the surface tension of the material surface and blood are different, an interface is formed; to reduce the interfacial tension, a certain driving force must be generated to cause an active component of the blood to move toward the interface [36]. Moacania et al. [37] measured the surface tension of more than 190 types of biological materials and found that coagulation on the surface of the biological materials was related to the dispersion and polar forces of the material surface. Michiardi [38] indicated that when the adsorption capacity of albumin on the material surface had a positive linear correlation with the polar component of its surface energy, a higher polar component resulted in a higher adsorption capacity of albumin; the adsorption of albumin was beneficial in reducing platelet adhesion. After MAO processing, the polar force component of the coating surface increased significantly, which improved the preferential adsorption of albumin, inhibited platelet adhesion, and enhanced blood compatibility.

MAO processing significantly enhanced the blood compatibility of ultrafine-grained pure titanium; it more effectively inhibited platelet adhesion, release, and aggregation, and reduced the occurrence of coagulation. This may be attributed to the fact that, after MAO processing, the porous anatase- and rutile-phase TiO_2_ ceramic coating containing Si, Ca, and P possessed smaller contact angles for distilled water, smaller work function, and a more suitable surface energy (higher polar force components of the surface energy), which changed the charge density, chemical composition, and other properties. Thus, the adsorption of albumin and fibrin on its surface was affected, further improving blood compatibility. Among these, the anticoagulant performance of coating with an oxidation time of 9 min was the most favorable, potentially because its hydrophilicity was the most favorable, its surface energy was the highest, and its polar force component was the largest. This caused the coating to adsorb more albumin and less fibrin and thus enhanced blood compatibility [39].

## 4. Conclusions

(1) MAO technology can cause in situ oxidation of the ultrafine-grained pure titanium surface to generate a porous coating with mixed crystal structures of anatase- and rutile-phase TiO_2_.
(2) After MAO modification, the samples with rough surface possessed smaller contact angles, higher surface energy, and more favorable wettability. The change in surface energy was primarily due to the increase of its polar force component. This was caused by how, after modification, the specific surface area of the MAO coating of the ultrafine-grained pure titanium increased, roughness increased, and the −OH^−^ and −O^2−^ oxygen-containing groups formed on the coating surface effectively introduced active hydrophilic groups.
(3) In comparison to the ultrafine-grained pure titanium substrate, MAO coating reduced the hemolysis rate; extended the dynamic coagulation time, PT, and APTT; reduced the amount of platelet adhesion and degree of deformation; and increased the anticoagulant property. In particular, the sample with an oxidation time of 9 min had the lowest hemolysis rate; the longest dynamic coagulation time, PT, and APTT; the most favorable anticoagulant property; and the least platelet adhesion, with no aggregation generated and the lowest degree of deformation.
(4) MAO is an ideal surface modification technique that can significantly enhance the blood compatibility of ultrafine-grained pure titanium. After MAO processing, the porous anatase- and rutile-phase TiO_2_ ceramic coating containing Si, Ca, and P possessed smaller contact angles for distilled water, smaller work function, and a more suitable surface energy (higher polar force components of the surface energy). This study showed that ultrafine-grained pure titanium modified by MAO possessed favorable blood compatibility, was in accordance with ISO requirements for medical materials, and has potential for use as a vascular stent material. However, further research is required on the short- and long-term effects of its implantation in the human body.

## Figures and Tables

**Table 1:** Elemental content of micro-arc oxidation coatings (at %).

Elements	E03	E06	E09	E12	E15
O	75.42	76.05	77.44	77.50	76.37
Si	2.66	4.04	3.31	3.46	3.97
P	0.93	1.71	1.73	1.79	2.27
Ca	0.93	2.17	2.94	3.20	4.50
Ti	20.06	15.01	15.20	14.04	12.89

**Table 2:** Surface energy parameters of test liquids.

Samples	$\gamma_{L}/\mathrm{mJ}\cdot m^{-2}$	$\gamma_{L}^{D}/\mathrm{mJ}\cdot m^{-2}$	$\gamma_{L}^{P}/\mathrm{mJ}\cdot m^{-2}$
Distilled water	72.1	19.9	52.2
Ethylene glycol	48.0	29.0	19.0

**Table 3:** PT (prothrombin time) and APTT (activated partial thromboplastin time) of samples.

Samples	E00	E03	E06	E09	E12	E15	Positive
PT	11.87 ± 0.15	11.93 ± 0.19	12.09 ± 0.16	13.72 ± 0.73	13.46 ± 0.0.59	12.60 ± 0.53	12.08 ± 0.23
APTT	26.5 ± 0.10	29.6 ± 0.32	31.8 ± 0.42	36.0 ± 0.21	30.9 ± 0.25	27.8 ± 0.38	28 ± 0.26
